# Limited cancer health literacy rates and factors associated with cancer health literacy in persons with or without a cancer diagnosis

**DOI:** 10.1080/28355245.2026.2641290

**Published:** 2026-03-17

**Authors:** Levent Dumenci, Danial l. Riddle

**Affiliations:** a Temple University, Philadelphia, Pennsylvania, USA; b Fox Chase Cancer Center, Philadelphia, Pennsylvania, USA; c Virginia Commonwealth University, Richmond, Virginia, USA

**Keywords:** Cancer health literacy, racial disparities, the CHLT-6, demographic characteristics

## Abstract

**Background:**

Health literacy is a key factor for health communication efforts. This is especially true for preventing noncommunicable chronic diseases such as cancer. Specifically, limited cancer health literacy (LCHL) is a risk factor for not engaging in disease prevention and treatment.

**Aims:**

This study aimed to obtain model-based estimates of LCHL rates and explore rate differences in sociodemographic characteristics.

**Methods:**

Data from cancer patients (N=1,306) and those without cancer (N=512) collected from the U.S. were used to estimate crude and adjusted LCHL rates using latent class analysis. Measurement invariance tests were conducted between demographic groups to test the assumption that LCHL has the same meaning across subpopulations.

**Results:**

Scalar invariance model was supported in all comparisons. Among cancer patients, approximately 10-fold crude rate differences in LCHL were found between Blacks (50%) and Whites (5%), patients with education level up to high school (54%) and above high school (6%), as well as patients earning less than $40K (50%) and $40K or above (4%) annually. A parallel 10-fold difference between race, education, and income groups was also found among persons without cancer but the LCHL rates were somewhat higher among persons without cancer than those with cancer across all groups.

**Discussion:**

Adjusted rate differences between groups were quite similar. LCHL is highly prevalent among Blacks, under-educated, and low-income persons with and without a diagnosis of cancer. Race, educational attainment, and income are the primary drivers of disparities in cancer health literacy. Our findings help to inform targeted health communication efforts to minimize adverse health and financial consequences of LCHL.

## PLAIN LANGUAGE SUMMARY

Identification of people with limited cancer health literacy (LCHL) helps to accurately estimate rates in populations with and without cancer, design effective interventions and test their effects, and eventually improve cancer care outcomes. We identified people with LCHL using scientifically sound methods and, stratified by cancer and non-cancer status, estimated LCHL rates in race, education, income, sex, and age groups. Blacks, low-income and low-education subpopulations had LCHL rates approximately ten-fold higher than Whites, high-education, and high-income subpopulations. Unadjusted and adjusted rates were similar in both subpopulations with and without cancer. In our view, the CHLT-6 can be used by care teams to screen for LCHL and inform discussions with patients and at the public health level to guide development of national efforts to develop interventions to reduce the impact of LCHL.

## Introduction

Limited health literacy (LHL) has become a worldwide concern as it hampers efforts to improve the outcomes of health interventions and increase the health cost (Kindig et al., 2004; Nutbeam & Lloyd, 2021; Santos et al., 2017). The estimated cost of LHL is up to $283 billion annually in the U.S (Vernon et al., 2007). Despite the importance of quantifying LHL rates, uncertainty in LHL rates is substantial. Five meta-analytic studies from the U.S, European, and Southeast Asian populations indicated that LHL rates cover nearly the entire plausible range: 0%–99.5% (Baccolini et al., 2021; Hamzah et al., 2016; Nilnate, 2014; Paasche‐Orlow et al., 2005; Rajah et al., 2019). The extent of uncertainty surrounding LHL rates limits sound public health and health policy decision making. For persons with cancer, limited cancer health literacy (LCHL) is likely even more consequential given the extensive morbidity and mortality and the consequences of non-adherence to treatment which may in part, be due to LCHL.

Most literature on health literacy relies on cut points along a continuum to identify individuals with LHL. These cut points are then used to compare health literacy rates between various groups. Except for studies using the Cancer Health Literacy Test-6 (CHLT-6; Dumenci et al., 2014), all studies reporting LHL rates have used an arbitrary cut-score to determine if a person has LHL by selecting a number (i.e. cut-score) along a continuum. The LHL label is assigned when a person’s score is below (or above) the cut-score. Further, comparisons of LHL rates assume that LHL has the same meaning for all groups (e.g. Blacks vs. Whites). This assumption remains untested when a cut-score method is used to determine LHL. The cut-score method of estimating prevalence rates was dismissed as non-scientific and even misleading by methodologists (Dumenci, 2011; Rindskopf & Rindskopf, 1986; Templin & Henson, 2010). Due to its convenience, however, it remains the most dominant method to determine LHL in extant literature. In the absence of scientifically determining who has LHL and who does not, any inferences about factors contributing to the differences in LHL rates remain premature when using cut scores. The target population is also a critical consideration in estimating LHL rates. Whereas the LHL rates estimated from persons without cancer are informative for designing targeted educational interventions, LHL rates estimated from populations with confirmed cases of cancer are needed to customize intervention efforts.

Prior studies consistently showed that health literacy is associated with race, gender, educational attainment, income, and age with varying degree across settings and populations. Racial and ethnic disparities have been broadly documented with ethnic minorities, Non-Hispanic Blacks in particular, topping the chart in LHL (Berkman et al., 2011; Coughlin et al., 2020; Paasche‐Orlow et al., 2005; Stormacq et al., 2019). Also, education has been found as one of the primary contributing factors to LHL (Shah et al., 2010; Sørensen et al., 2015; Weiss, 2007). This is no surprise as health literacy is subsumed under general literacy, which increases with educational attainment level. It has been reported that education and race remain significant predictors of LHL after adjusting for covariates in the meta-analysis of U.S. studies (Paasche‐Orlow et al., 2005). Income, age, and gender have also been repeatedly found as contributing factors in health literacy disparities in two meta-analytic studies outside the U.S. (Baccolini et al., 2021; Rajah et al., 2019).

An instrument specific to cancer is useful because of the complex treatment choices patients face, the substantial morbidity associated with treatment adherence and non-adherence, along with the increased demand for self-care. Given the extensive human suffering and other costs of cancer diagnosis and care, a health literacy instrument designed for patients with cancer appears to be needed. Other chronic illnesses that require an engaged patient to assure optimal outcomes via adherence to sometimes challenging treatment regimens, such as diabetes and hypertension, may also warrant specific health literacy measures. Treatment adherence is even more challenging with cancer, given known side effects of many cancer treatments. Individual characteristics contributing to limited cancer health literacy (LCHL) rates and differences in these rates for persons with cancer and those without cancer are unknown.

The primary aim of this study was twofold: To estimate LCHL rates using model-based approaches and to quantify LCHL rate differences associated with disparities in LCHL. We hypothesized that LCHL rates differ in educational attainment, race, income, age, and gender groups, as consistent with the extant literature on general health literacy, and that patients with cancer have lower rates of LCHL than those without cancer.

## Methods

### Study design and participants

As a part of the Cancer Health Literacy Study, data were collected from a National Institute of Cancer designated cancer institute at Mid-Atlantic region, and affiliated clinics (persons with cancer diagnosis; *n* = 1306) and churches, primary care clinics, health fairs, and community centers (persons without cancer diagnosis; *n* = 512). All 1818 participants met the eligibility criteria: (a) ages 18 or older; (b) English-speaking, and (c) no physical or cognitive impairment to prevent completion of the study protocol.

A hand-held touchscreen device was used for test administration with the option of read-aloud for those with limited reading ability. A signed informed consent form was obtained from each participant prior to enrollment into the study. A detailed explanation of procedures has been reported elsewhere (Dumenci et al., 2014, 2018).

### Measures

The CHLT-6 is a six-item standardized instrument with multiple choice response format designed to identify persons with LCHL (Dumenci et al., 2014). When we developed the CHLT-6, we noted that there was controversy regarding whether health literacy was an ability, a skill, or knowledge. When we developed cancer health literacy instruments, we adopted an ability definition of health literacy. Consequently, our CHLT items include knowledge and skills, as well as items that require synthesizing knowledge and skills. A strong support for the measurement structure of CHLT-6 that we report in this study indicates that knowledge, skill, and their synthesis are highly correlated.

A discrete latent variable modeling, i.e. latent class analysis (LCA) with two classes [LCHL and Adequate Cancer Health Literacy (ACHL)], underlies the measurement structure of the instrument. The CHLT-6 has been developed from cancer patient populations (Dumenci et al., 2014) and has been validated in persons without cancer (Dumenci et al., 2018). Cross-cultural validation of the instrument has been reported in Chinese cancer populations from Hong Kong (Chan et al., 2022). Accuracy rates of the CHLT-6 for identifying persons with LCHL were between 90% and 95% and with ACHL between 94% and 96% (Chan et al., 2022; Dumenci et al., 2014, 2018). Conditional probabilities were used to define the classes. These probabilities were moderate for the LCHL class (range: 0.49–0.67) and very high for the ACHL class (range: 96%–99%) (Dumenci et al., 2014). Expected a posteriori probability (EAP) estimates are used for scoring. For a given item response pattern across six items, there was a unique probability of belonging to LCHL. The probability of belonging to ACHL was simply one minus the probability of belonging to LCHL. The CHLT-6 and scoring rules are provided in the Supplementary material.

### Statistical analysis

Prior to data analysis, educational level, income, and age were transformed into binary variables: Education: ≤high school versus > high school; annual income: <$40K versus ≥$40K, and age: <65 years versus ≥65 years. Stratified by cancer and non-cancer status, and the measurement structure of the CHLT-6, a confirmatory LCA with two classes, was used to test the hypothesis that item response patterns are consistent with the two-class solutions for cancer and non-cancer groups for the following variables: Race (Black vs. White), education (≤high school vs. >high school), income (<$40K vs. ≥$40K), age (<65 yrs. vs. ≥65 yrs.), and sex (male vs. female). The likelihood ratio chi-square test of model fit, entropy, and average most likely posterior probabilities were used to assess model fit and class separation. Low entropy values (<0.60) indicated poor class separation. High average most likely posterior probabilities (>0.90) is considered as strong evidence for class separation even when the entropy is low (Sinha et al., 2021). For each binary variable, measurement invariance was used to test the assumption that LCHL and ACHL labels have the same meaning between groups by testing the between group equality constraints on conditional probabilities. As the unconstrained (configural invariance) and between-group conditional probability constrained (scalar invariance) models were not nested, Bayesian Information Criterion (BIC) was used to select the best fitting model with smaller BIC indicating a better fit. The LCHL rate estimates (unconditional probabilities) were obtained from the measurement invariant model when the scalar invariance model was selected from the model comparisons. In addition to crude LCHL rates, the adjusted rates were obtained for each group after controlling for the remaining four demographic variables. Finally, the measurement invariance between cancer and non-cancer groups was conducted to test the assumption that LCHL and ACHL are defined the same way between patients with and without a cancer diagnosis. Methods recommended by Kankaraš et al. (2018) were used to test measurement invariance for our categorical data.

Overall, the missing item response rate was very low (0.14%). Missing item responses were handled using full information maximum likelihood method in all latent variable models using Mplus (v.9; Muthén & Muthén 1998–2025). Participants with missing grouping variable were excluded in the measurement invariance test as multi-group model specifications require an observed (i.e. non-missing) grouping variable.

## Results

### Participant characteristics

For all participants in the cancer group, the cancer diagnosis was confirmed by their oncologists, and all the participants in the non-cancer subgroup self-reported that they had no cancer diagnosis. Participant age ranged from 18 to 93 years. Sex, race (Blacks and Whites) and income (<$40K and ≥40K) were approximately equally distributed. One-third had no more than a high school degree. Low- and high-income groups were approximately equally distributed in both cancer and non-cancer groups. Table 1 lists the demographic characteristics for patients with versus without a cancer diagnosis.

### Measurement structure and precision

Stratified by presence or absence of a cancer diagnosis, race, education, income, age, and gender groups, model fit and class separation indices from LCA with two-classes were listed in Table 2. Results indicated that the CHLT-6 clearly and precisely separated LCHL and ACHL classes with high precision, i.e. very high average latent class probabilities for the most likely class: LCHL range: 0.79–0.99; ACHL range: 0.91–0.99.

### Measurement invariance

Results from the cancer diagnosis (present or absent) stratified measurement invariance tests appear in Table 3. For race, education, income, age, and gender groups, the conditional probability constrained model (scalar invariance) fit as good as or better than the configural invariance model (i.e. BIC for the scalar invariance model < configural invariance) indicating that the LCHL and ACHL labels have the same meaning between the groups for all five participant characteristics.

### The LCHL rates

**Figure 1.**
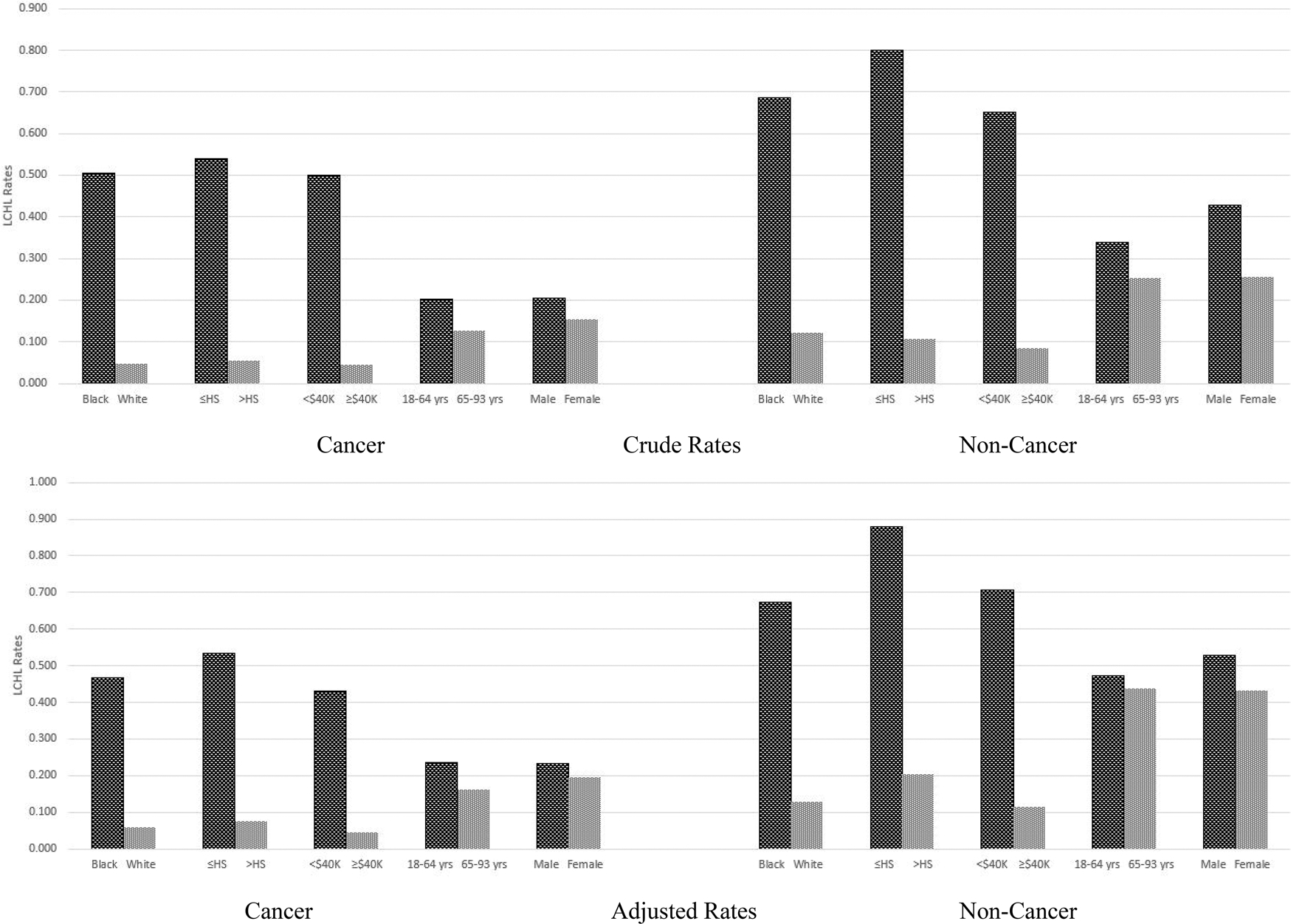
Limited cancer health literacy rates.

Stratified by cancer and noncancer groups, crude and adjusted unconditional probability estimates (i.e. the LCHL rates) from the scalar invariance model are provided in Figure 1. Among persons with cancer diagnosis, LCHL rates are 4%–6% for persons self-reporting as Caucasian or White, persons above high school education and annual income at or above $40K. LCHL rates are reported for persons self-reporting as African American or Black, persons with high school or less educational attainment, and making less than $40K were 50%–54%. Patterns of results are similar among patients without cancer with somewhat elevated LCHL rates as compared to persons with a cancer diagnosis. Among persons without cancer diagnosis, LCHL rates are 8%–12% for Whites, persons above high school education and income above $40K. LCHL rates are similar for persons self-reporting as Black/African American, persons with high school or low educational attainment, and making less than $40K were 65%–80%. In both cancer and non-cancer samples, younger adults (18–64 years) have slightly higher rates of LCHL than older adults (65–93 years). Similarly, males have somewhat higher rates of LCHL than females. Race, education, and income group differences remain stable after covariate adjustments, but somewhat small sex and age group differences further diminished with adjustments in both cancer and non-cancer samples. Blacks consistently have higher rates of LCHL than Whites at each and every level of income and educational attainment.

### Cancer versus non-cancer comparisons

**Figure 2.**
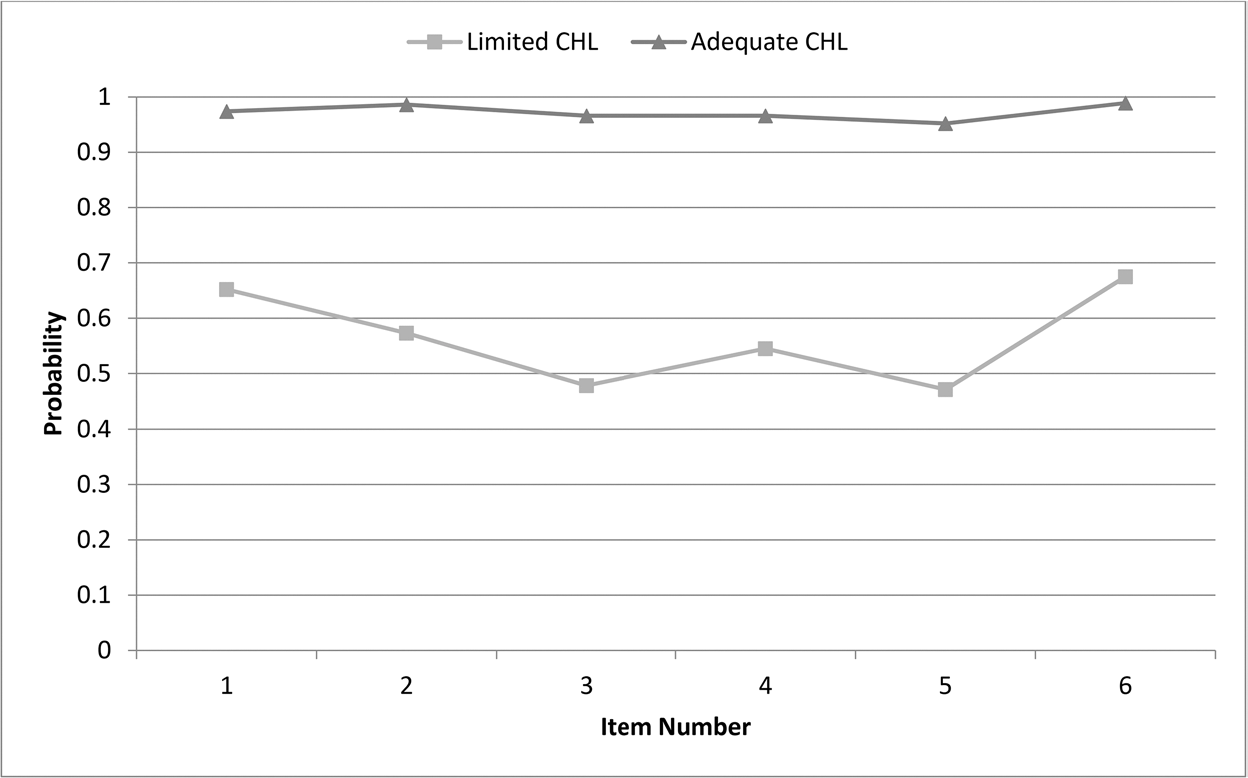
Conditional probability estimates from latent class analysis of the CHLT-6 with measurement invariance between cancer and non-cancer samples.

The measurement invariance test between cancer and non-cancer groups was supported with a BIC value from scalar invariance lower than the configural invariance (BIC: 9117 vs. 9167). Conditional probability estimates from the scalar invariance model (i.e. the model-based definitions of LCHL and ACHL) appear in Figure 2. Persons from the LCHL class have a probability of correct item response slightly higher than the chance level (range: 0.48–0.68), and those in the ACHL class have a very high probability of answering all six items correctly (range: 0.95–0.99).

## Discussion

Our hypothesized group differences in LCHL rates were supported for participants diagnosed with cancer and participants without a cancer diagnosis. Our results indicated that race, education and income are the primary factors contributing to differences in LCHL rates. For Blacks, persons with education level no more than high school, and income below $40K had very high rates of LCHL ranging from 50% to 63%, as compared to White persons, education above high school and income at least $40K having LCHL rates no more than 7% among persons with cancer. We also found higher LCHL rates for persons without versus persons with a cancer diagnosis, younger versus older adults, and males versus females yet the group differences were not as striking as the differences in race, educational attainment, and income groups. The pattern of results was similar for patients without cancer, but the LCHL rates are somewhat elevated compared to persons with cancer.

Relationships between cancer health literacy and race, educational attainment, income, age, and gender in our study have been reported in prior studies using general health literacy instruments (Paasche‐Orlow et al., 2005; Rajah et al., 2019; Shah et al., 2010; Sørensen et al., 2015; Weiss, 2007). In addition to generalizing results to specifically cancer health literacy across cancer and non-cancer populations, our study was unique in determining the LCHL using a model-based approach to classification. That is, the classification error is accounted for in LCHL rate estimates. Further, we statistically tested the lexical equivalence of descriptive labels (LCHL and ACHL) between groups prior to comparing them. Considerable efforts have been underway to make latent mixture modeling, the LCA in particular, accessible to applied public health researchers (Aflaki et al., 2022; Eshima, 2022; Nylund-Gibson & Choi, 2018; Porcu & Giambona, 2017).

From the public health perspective, LCHL rates estimated from those without confirmed cancer diagnosis are essential in designing targeted health education interventions. Our study showed that the CHLT-6 had invariant measurement properties between cancer and non-cancer populations and that persons without a cancer diagnosis have somewhat higher rates of LCHL across all groups. In addition to calls for health communication campaigns to increase health literacy levels of patients, future studies are needed to investigate the compensatory effects of caregivers’ cancer health literacy levels on cancer patients’ cancer health literacy so that health education activities could be extended to those without a cancer diagnosis to help improve health outcomes and reduce health costs. At the individual patient level, patients waiting to see their doctor can complete CHLT-6 in just a few minutes. Seeing these results in advance allows the care team to prepare their discussions with patients to be more understandable.

### Strengths and limitations

The major strength of this study is the use of a model-based method to determine if a cancer patient or a person without cancer has LCHL. Further, the LCHL rates between groups were compared once it was established that the LCHL label had the same meaning between groups to ensure that it is an equivalent comparison. Specifically, all rates reported in this study were estimated from the scalar invariance models. Also, the LCHL rates were estimated separately for those with and without a confirmed cancer diagnosis to help inform primary, secondary and tertiary health interventions.

Limitations of this study warrant discussion. No claim could be made about the samples being representative of the entire U.S. population. For example, the samples included Blacks and Whites and no other racial/ethnic groups, which limits the generalizability of our results and all data were collected from one site in the southeast US. Also, age, education, and income are inherently continuous variables. Different grouping rules would inevitably yield different LCHL rates.

## Conclusion

Race, education, and income are the primary predictors of health disparities in cancer health literacy. Sex and age have considerably smaller associations with cancer health literacy as compared to race, education, and income. Health professionals are urged to identify individuals with LCHL early on and take concerted efforts to reduce disparities and potentially improve cancer care outcomes and the financial burden of LCHL. Providers should be especially cognizant of critical, yet unmodifiable patient characteristics such as race, and potentially modifiable characteristics such as education and take a proactive role in addressing cancer health literacy needs in clinical care settings, for example, through educationally grounded interventions designed to improve LCHL.

## Supplementary Material

Supp 1

Supplemental data for this article can be accessed online at https://doi.org/10.1080/28355245.2026.2641290.

## Figures and Tables

**Table 1.** Participant characteristics.

Variable	Cancer (*N* = 1306)			Non-cancer (*N* = 512)		
	*N*	%	Missing	*N*	%	Missing

Age	896	68.61	0	444	87.06	2
Younger adults (18–64 yrs)	410	31.39		66	12.94	
Older adults (65–93 yrs)						
Sex	716	54.82	0	315	61.52	30
Female	590	45.18		197	38.48	
Male						
Race	492	37.39	0	328	64.01	0
Black	806	61.25		180	35.16	
White	8	1.36		4	0.83	
Other						
Education			1			0
High school or less	397	30.42		205	40.04	
Higher than high school	908	69.58		307	59.96	
Income	516	43.88	130	263	59.50	70
Low (Less than $40,000)	660	56.12		179	40.50	
High ($40,000 or more)						

**Table 2.** Latent class analysis with two classes.

Diagnosis	Variable	Group	LRT *χ*^2^_*(df*=50*)*_	*p*	Entropy	Accuracy^a^	
						LCHL	ACHL

Cancer:	Race/Ethnicity	Black	76.17	0.010	0.691	0.886	0.939
		White	50.57	0.451	0.874	0.933	0.977
	Education	≤High School	49.33	0.002	0.667	0.910	0.917
		>High School	43.31	0.737	0.871	0.929	0.976
	Income	<$40K	78.65	0.006	0.740	0.930	0.940
		≥$40K	29.79	0.990	0.772	0.789	0.956
	Age (years)	<65	76.51	0.009	0.824	0.938	0.965
		≥65	52.91	0.362	0.850	0.951	0.969
	Sex	Female	79.84	0.005	0.824	0.950	0.963
		Male	43.28	0.738	0.836	0.939	0.967
Non-cancer	Race/Ethnicity	Black	38.66	0.878	0.610	0.851	0.910
		White	38.84	0.874	0.922	0.982	0.983
	Education	≤High School	48.29	0.542	0.502	0.875	0.828
		>High School	46.43	0.618	0.767	0.908	0.951
	Income	<$40K	45.62	0.642	0.625	0.819	0.943
		≥$40K	30.68	0.986	0.906	0.991	0.976
	Age (years)	<65	45.66	0.648	0.736	0.917	0.942
		≥65	36.91	0.916	0.781	0.931	0.994
	Sex	Female	49.69	0.486	0.714	0.874	0.946
		Male	61.83	0.112	0.784	0.965	0.935

LRT: Likelihood ratio test.

a Average latent class probabilities for the most likely class membership.

**Table 3.** Measurement invariance tests for the CHLT-6.

Diagnosis	Variable	Groups	Configural			Scalar		
			LRT *χ*^2^_*(df=100)*_	*p*	*BIC*	LRT *χ*^2^_*(df=112)*_	*p*	*BIC*

Cancer:	Race/Ethnicity	Black vs. White	126.75	0.037	5776	162.18	0.001	5725
	Education	≤HS vs. >HS	132.44	0.017	5681	153.31	0.005	5616
	Income	<$40K vs. ≥$40K	100.44	0.265	5180	145.45	0.018	5132
	Age	Male vs. Female	129.42	0.026	5967	151.24	<0.001	5903
	Sex	White vs. Black	123.11	0.058	6156	135.47	0.065	6002
Non-cancer:	Race/Ethnicity	Black vs. White	77.50	0.954	3293	126.50	0.165	3268
	Education	≤HS vs. >HS	94.72	0.630	3277	113.77	0.436	3221
	Income	<$40K vs. ≥$40K	72.58	0.982	2795	113.94	0.431	2759
	Age	Male vs. Female	82.57	0.897	3142	108.70	0.571	3094
	Sex	White vs. Black	111.51	0.203	3444	126.99	0.158	3385

HS: High school; LRT: Likelihood ration test; BIC: Bayesian Information Criterion.

## Data Availability

The data that support the findings of this study are available from the corresponding author upon reasonable request.
