# Supplementary material for: Limited cancer health literacy rates and factors associated with cancer health literacy in persons with or without a cancer diagnosis
Source: Health Lit Commun Open. Author manuscript; Available in PMC 2026 Apr 9. (PMC13059927; doi:10.1080/28355245.2026.2641290)
Supplement: Supp 1 [file NIHMS2157340-supplement-Supp_1.docx]

Supplementary Materials

1. Cancer Health Literacy Test – 6:

Instruction: This test contains items with correct and incorrect answers. For each test item, please select only one answer that you believe to be correct by circling the letter. Complete the test by answering all items.

1) The normal range for hemoglobin for a male is 13.3 – 17.2 g/dl. Joe’s hemoglobin is 9.7 g/dl. Is Joe within the normal range?

- 1. Yes
  2. No

1. A biopsy of a tumor is done to:
   1. Remove it
   2. Diagnose it
   3. Treat it
2. If a patient has stage 1 cancer, it means the cancer is:
   1. Localized
   2. In nearby organs
   3. In distant sites
3. The role of a physical therapist is to talk to a patient about emotional needs.
   1. True
   2. False
4. A tumor is considered “inoperable” when it cannot be treated with:

a) Radiation Therapy

b) Surgery

c) Chemotherapy

1. Sally will get radiation therapy once a day, Monday through Friday. If Sally has therapy for 4 weeks, how many times will she get radiation therapy?
   1. 5
   2. 15
   3. 20

Copyright 2014 L. Dumenci. All rights reserved.

1. CHLT-6 Scoring

|  |  |  |  |  |  |  | Probability of | | Probability of |
| --- | --- | --- | --- | --- | --- | --- | --- | --- | --- |
| item-1 | item-2 | item-3 | item-4 | item-5 | item-6 |  | LCHL |  | ACHL |
| 0 | 0 | 0 | 0 | 0 | 0 |  | > 0.99 |  | < 0.01 |
| 0 | 0 | 0 | 0 | 0 | 1 |  | > 0.99 |  | < 0.01 |
| 0 | 0 | 0 | 0 | 1 | 0 |  | > 0.99 |  | < 0.01 |
| 0 | 0 | 0 | 0 | 1 | 1 |  | > 0.99 |  | < 0.01 |
| 0 | 0 | 0 | 1 | 0 | 0 |  | > 0.99 |  | < 0.01 |
| 0 | 0 | 0 | 1 | 0 | 1 |  | > 0.99 |  | < 0.01 |
| 0 | 0 | 0 | 1 | 1 | 0 |  | > 0.99 |  | < 0.01 |
| 0 | 0 | 0 | 1 | 1 | 1 |  | > 0.99 |  | < 0.01 |
| 0 | 0 | 1 | 0 | 0 | 0 |  | > 0.99 |  | < 0.01 |
| 0 | 0 | 1 | 0 | 0 | 1 |  | 0.88 |  | 0.12 |
| 0 | 0 | 1 | 0 | 1 | 0 |  | > 0.99 |  | < 0.01 |
| 0 | 0 | 1 | 0 | 1 | 1 |  | > 0.99 |  | < 0.01 |
| 0 | 0 | 1 | 1 | 0 | 0 |  | > 0.99 |  | < 0.01 |
| 0 | 0 | 1 | 1 | 0 | 1 |  | > 0.99 |  | < 0.01 |
| 0 | 0 | 1 | 1 | 1 | 0 |  | > 0.99 |  | < 0.01 |
| 0 | 0 | 1 | 1 | 1 | 1 |  | 0.92 |  | 0.08 |
| 0 | 1 | 0 | 0 | 0 | 0 |  | > 0.99 |  | < 0.01 |
| 0 | 1 | 0 | 0 | 0 | 1 |  | > 0.99 |  | < 0.01 |
| 0 | 1 | 0 | 0 | 1 | 0 |  | > 0.99 |  | < 0.01 |
| 0 | 1 | 0 | 0 | 1 | 1 |  | > 0.99 |  | < 0.01 |
| 0 | 1 | 0 | 1 | 0 | 0 |  | > 0.99 |  | < 0.01 |
| 0 | 1 | 0 | 1 | 0 | 1 |  | > 0.99 |  | < 0.01 |
| 0 | 1 | 0 | 1 | 1 | 0 |  | > 0.99 |  | < 0.01 |
| 0 | 1 | 0 | 1 | 1 | 1 |  | 0.87 |  | 0.13 |
| 0 | 1 | 1 | 0 | 0 | 0 |  | > 0.99 |  | < 0.01 |
| 0 | 1 | 1 | 0 | 0 | 1 |  | > 0.99 |  | < 0.01 |
| 0 | 1 | 1 | 0 | 1 | 0 |  | > 0.99 |  | < 0.01 |
| 0 | 1 | 1 | 0 | 1 | 1 |  | 0.84 |  | 0.16 |
| 0 | 1 | 1 | 1 | 0 | 0 |  | > 0.99 |  | < 0.01 |
| 0 | 1 | 1 | 1 | 0 | 1 |  | 0.84 |  | 0.16 |
| 0 | 1 | 1 | 1 | 1 | 0 |  | 0.92 |  | 0.08 |
| 0 | 1 | 1 | 1 | 1 | 1 |  | 0.16 |  | 0.84 |
| 1 | 0 | 0 | 0 | 0 | 0 |  | > 0.99 |  | < 0.01 |
| 1 | 0 | 0 | 0 | 0 | 1 |  | > 0.99 |  | < 0.01 |
| 1 | 0 | 0 | 0 | 1 | 0 |  | > 0.99 |  | < 0.01 |
| 1 | 0 | 0 | 0 | 1 | 1 |  | > 0.99 |  | < 0.01 |
| 1 | 0 | 0 | 1 | 0 | 0 |  | > 0.99 |  | < 0.01 |
| 1 | 0 | 0 | 1 | 0 | 1 |  | > 0.99 |  | < 0.01 |
| 1 | 0 | 0 | 1 | 1 | 0 |  | > 0.99 |  | < 0.01 |
| 1 | 0 | 0 | 1 | 1 | 1 |  | 0.94 |  | 0.06 |
| 1 | 0 | 1 | 0 | 0 | 0 |  | > 0.99 |  | < 0.01 |
| 1 | 0 | 1 | 0 | 0 | 1 |  | > 0.99 |  | < 0.01 |
| 1 | 0 | 1 | 0 | 1 | 0 |  | > 0.99 |  | < 0.01 |
| 1 | 0 | 1 | 0 | 1 | 1 |  | 0.93 |  | 0.07 |
| 1 | 0 | 1 | 1 | 0 | 0 |  | > 0.99 |  | < 0.01 |
| 1 | 0 | 1 | 1 | 0 | 1 |  | 0.93 |  | 0.07 |
| 1 | 0 | 1 | 1 | 1 | 0 |  | 0.97 |  | 0.03 |
| 1 | 0 | 1 | 1 | 1 | 1 |  | 0.31 |  | 0.69 |
| 1 | 1 | 0 | 0 | 0 | 0 |  | > 0.99 |  | < 0.01 |
| 1 | 1 | 0 | 0 | 0 | 1 |  | > 0.99 |  | < 0.01 |
| 1 | 1 | 0 | 0 | 1 | 0 |  | > 0.99 |  | < 0.01 |
| 1 | 1 | 0 | 0 | 1 | 1 |  | 0.88 |  | 0.12 |
| 1 | 1 | 0 | 1 | 0 | 0 |  | > 0.99 |  | < 0.01 |
| 1 | 1 | 0 | 1 | 0 | 1 |  | 0.88 |  | 0.12 |
| 1 | 1 | 0 | 1 | 1 | 0 |  | 0.94 |  | 0.06 |
| 1 | 1 | 0 | 1 | 1 | 1 |  | 0.21 |  | 0.79 |
| 1 | 1 | 1 | 0 | 0 | 0 |  | > 0.99 |  | < 0.01 |
| 1 | 1 | 1 | 0 | 0 | 1 |  | 0.85 |  | 0.15 |
| 1 | 1 | 1 | 0 | 1 | 0 |  | 0.93 |  | 0.07 |
| 1 | 1 | 1 | 0 | 1 | 1 |  | 0.17 |  | 0.83 |
| 1 | 1 | 1 | 1 | 0 | 0 |  | 0.93 |  | 0.07 |
| 1 | 1 | 1 | 1 | 0 | 1 |  | 0.18 |  | 0.82 |
| 1 | 1 | 1 | 1 | 1 | 0 |  | 0.31 |  | 0.69 |
| 1 | 1 | 1 | 1 | 1 | 1 |  | < 0.01 |  | > 0.99 |

Table 1. Participant characteristics

|  | Cancer (N=1,306) | | | | Non-Cancer (N=512) | | |
| --- | --- | --- | --- | --- | --- | --- | --- |
| Variable | N | % | | Missing | N | % | Missing |
| Age  Younger adults (18 – 64 yrs)  Older adults (65 – 93 yrs) | 896  410 | 68.61  31.39 | | 0 | 444  66 | 87.06  12.94 | 2 |
| Sex  Female  Male | 716  590 | | 54.82  45.18 | 0 | 315  197 | 61.52  38.48 | 30 |
| Race  Black  White  Other | 492  806  8 | 37.39  61.25  1.36 | | 0 | 328  180  4 | 64.01  35.16  0.83 | 0 |
| Education |  |  | | 1 |  |  | 0 |
| High school or less | 397 | 30.42 | |  | 205 | 40.04 |  |
| Higher than high school | 908 | 69.58 | |  | 307 | 59.96 |  |
|  |  |  | |  |  |  |  |
| Income  Low (Less than $40,000)  High ($40,000 or more) | 516  660 | 43.88  56.12 | | 130 | 263  179 | 59.50  40.50 | 70 |

Table 2. Latent class analysis with two classes

| Diagnosis | Variable | Group | LRT $\chi^{2}$*_(df_*_=50_*_)_* | *p* | Entropy | Accuracy^1^ | |
| --- | --- | --- | --- | --- | --- | --- | --- |
|  |  |  |  |  |  | LCHL | ACHL |
| Cancer: | Race/Ethnicity | Black | 76.17 | .010 | .691 | 0.886 | 0.939 |
|  |  | White | 50.57 | .451 | .874 | 0.933 | 0.977 |
|  | Education | ≤High School | 49.33 | .002 | .667 | 0.910 | 0.917 |
|  |  | >High School | 43.31 | .737 | .871 | 0.929 | 0.976 |
|  | Income | <$40K | 78.65 | .006 | .740 | 0.930 | 0.940 |
|  |  | ≥$40K | 29.79 | .990 | .772 | 0.789 | 0.956 |
|  | Age (years) | <65 | 76.51 | .009 | .824 | 0.938 | 0.965 |
|  |  | ≥65 | 52.91 | .362 | .850 | 0.951 | 0.969 |
|  | Sex | Female | 79.84 | .005 | .824 | 0.950 | 0.963 |
|  |  | Male | 43.28 | .738 | .836 | 0.939 | 0.967 |
|  |  |  |  |  |  |  |  |
| Non-cancer | Race/Ethnicity | Black | 38.66 | .878 | .610 | 0.851 | 0.910 |
|  |  | White | 38.84 | .874 | .922 | 0.982 | 0.983 |
|  | Education | ≤High School | 48.29 | .542 | .502 | 0.875 | 0.828 |
|  |  | >High School | 46.43 | .618 | .767 | 0.908 | 0.951 |
|  | Income | <$40K | 45.62 | .642 | .625 | 0.819 | 0.943 |
|  |  | ≥$40K | 30.68 | .986 | .906 | 0.991 | 0.976 |
|  | Age (years) | <65 | 45.66 | .648 | .736 | 0.917 | 0.942 |
|  |  | ≥65 | 36.91 | .916 | .781 | 0.931 | 0.994 |
|  | Sex | Female | 49.69 | .486 | .714 | 0.874 | 0.946 |
|  |  | Male | 61.83 | .112 | .784 | 0.965 | 0.935 |

Note: LRT: Likelihood ratio test

^1^Average latent class probabilities for the most likely class membership.

Table 3. Measurement invariance tests for the CHLT-6

| Diagnosis | Variable | Groups | Configural | | | Scalar | | |
| --- | --- | --- | --- | --- | --- | --- | --- | --- |
|  |  |  | LRT$\chi^{2}$*_(df=100)_* | *p* | *BIC* | LRT$\chi^{2}$*_(df=112)_* | *p* | *BIC* |
| Cancer: | Race/Ethnicity | Black vs. White | 126.75 | 0.037 | 5776 | 162.18 | 0.001 | 5725 |
|  | Education | ≤HS vs. >HS | 132.44 | 0.017 | 5681 | 153.31 | 0.005 | 5616 |
|  | Income | <$40K vs. ≥$40K | 100.44 | 0.265 | 5180 | 145.45 | 0.018 | 5132 |
|  | Age | Male vs. Female | 129.42 | 0.026 | 5967 | 151.24 | <0.001 | 5903 |
|  | Sex | White vs. Black | 123.11 | 0.058 | 6156 | 135.47 | 0.065 | 6002 |
| Non-cancer: | Race/Ethnicity | Black vs. White | 77.50 | 0.954 | 3293 | 126.50 | 0.165 | 3268 |
|  | Education | ≤HS vs. >HS | 94.72 | 0.630 | 3277 | 113.77 | 0.436 | 3221 |
|  | Income | <$40K vs. ≥$40K | 72.58 | 0.982 | 2795 | 113.94 | 0.431 | 2759 |
|  | Age | Male vs. Female | 82.57 | 0.897 | 3142 | 108.70 | 0.571 | 3094 |
|  | Sex | White vs. Black | 111.51 | 0.203 | 3444 | 126.99 | 0.158 | 3385 |

Note: HS: High school; LRT: Likelihood ration test; BIC: Bayesian Information Criterion.


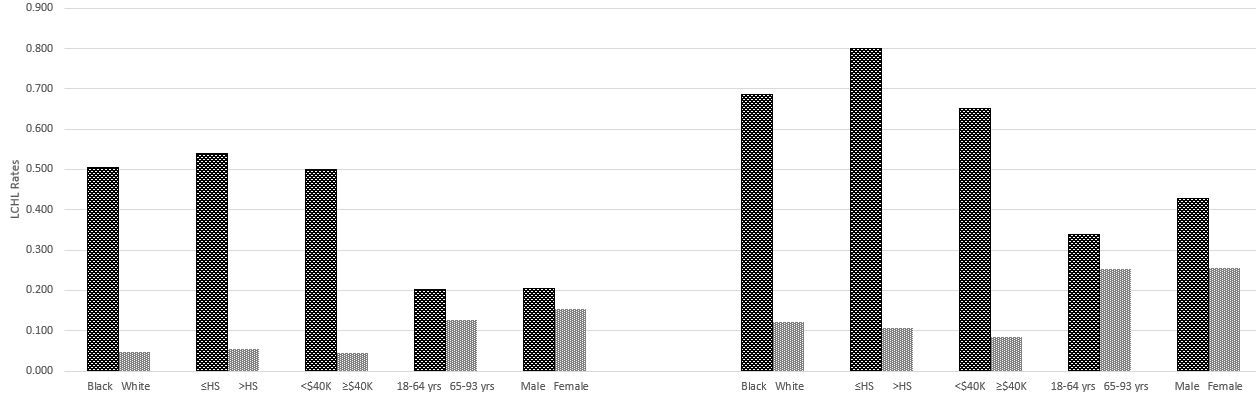


Cancer Crude Rates Non-Cancer


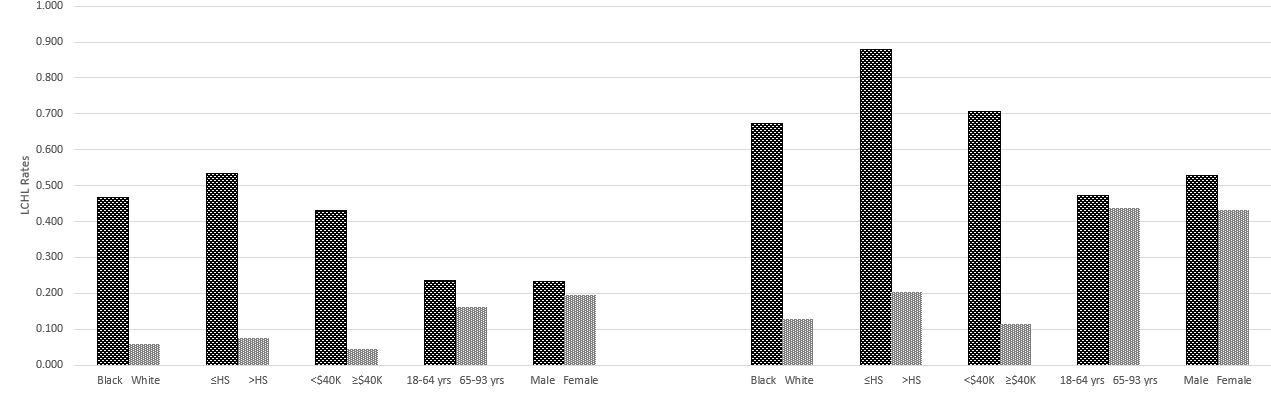


Cancer Adjusted Rates Non-Cancer
